# Risk of non-affective psychotic disorder and post-traumatic stress disorder by refugee status in Sweden

**DOI:** 10.1136/jech-2019-212798

**Published:** 2019-11-25

**Authors:** Arvinder K Duggal, James B Kirkbride, Christina Dalman, Anna-Clara Hollander

**Affiliations:** 1 Department of Public Health Sciences, Karolinska Institute, Stockholm, Sweden; 2 Division of Psychiatry, UCL, London, UK; 3 Division of Public Health Epidemiology, Department of Public Health Sciences, Karolinska Institutet, Stockholm, Sweden; 4 Psykisk Hälsa, Centrum för epidemiologi och samhällsmedicin, Stockholm, Sweden

**Keywords:** cohort studies, migration, psychiatry, public health, record linkage

## Abstract

**Background:**

Refugees have different experiences of obtaining a refugee status, however it remains unclear if this affects their risk of psychiatric disorders. The aim of this study was to investigate whether risk for non-affective psychotic disorder (NAPD) and post-traumatic stress disorder (PTSD) differs between quota refugees (resettled from refugee camps) and non-quota refugees (former asylum seekers).

**Method:**

A register-based cohort with a sample size of 52 561 refugees in Sweden starting 1 January 1997 ending 31 December 2011. Exposure: refugee status (quota or non-quota refugees). Cox regression models estimated adjusted HRs with 95% CIs for NAPD (International Classification of Diseases, Tenth Revision (ICD-10), F20–29) and PTSD (ICD-10, F43.1) by refugee status.

**Results:**

There were more non-quota refugees (77.0%) than quota refugees (23.0%). In total we identified 401 cases of NAPD, 1.0% among quota refugees and 0.7% among non-quota refugees, and 1070 cases of PTSD, 1.9% among quota refugees and 2.1% among non-quota refugees. Male quota refugees were at increased risk for NAPD compared with male non-quota refugees (HR_male_=1.41, 95% CI 1.09 to 1.82 and HR_female_=0.65, 95% CI 0.42 to 1.00). All quota refugees were at a reduced risk of PTSD compared with non-quota refugees (HR=0.74, 95% CI 0.64 to 0.87).

**Conclusions:**

This study suggests that risk of NAPD and PTSD varies for quota and non-quota refugees, highlighting the possibility that different experiences of the migration process differentiate the risk of psychiatric disorders among refugees.

## Introduction

Migration is a multistage process comprising distinct phases including premigration, migration and postmigration.^1^ Migration has been extensively studied as a risk factor for mental health conditions including schizophrenia and other non-affective psychotic disorders (NAPD).^2–7^ In 2018, the United Nations High Commissioner for Refugees (UNHCR) reported 68.5 million forcibly displaced people worldwide, which included 25.4 million refugees, the highest since records began.^8^ Growing research has revealed that refugees are an ultraexposed subgroup among migrants and are more vulnerable to psychiatric disorders,^4–7^ even when compared with migrants from the same region.^5 9^ Due to refugees’ high exposure to trauma^7^ the focus for studies on refugee mental health has often been post-traumatic stress disorder (PTSD),^4^ and more recently psychosis.^5^ There appear to be regional differences in the risk of PTSD, partly explained by the level of trauma the general population are exposed to, with women appearing to be at greater risk of developing PTSD than men.^10 11^ Refugees face an increased risk of psychosis compared with non-refugees, however regional differences do exist.^5^ In addition, the increased risk of psychosis among refugees is more apparent in men than in women.^5^

Refugees are often studied collectively as a migrant subgroup, however nuances exist within this group, in particular there are marked differences in their migration journey.^6 7^ To better understand what impact the different stages of the migration process have on the risk of PTSD and psychosis among refugees, we focus on two common but distinctly different types of refugees: *quota* and *non-quota refugees*. Quota refugees are defined as those who have undergone resettlement, defined by the UNHCR as ‘the selection and transfer of refugees from a State in which they have sought protection to a third State which has agreed to admit them—as refugees—with permanent residence status’.^8^ These refugees have often been forced to live in refugee camps or urban settings, for prolonged periods of time, close to the region of conflict, often in disadvantaged conditions, where they may have unmet health needs.^12^ In contrast, non-quota refugees have sought asylum at a country border and, based on the decision of their application for refugee status, have received a residence permit from that country. Unlike quota refugees, non-quota refugees have not experienced an organised resettlement programme, but may have made long, unsafe journeys in order to get a refugee status and may also have been subject to violations of their human rights during this period.^13^ Quota and non-quota are likely to have experienced different obstacles and stressors along their struggle to get to safety. Studies focusing on refugee camps have identified a high prevalence of mental disorders, disability and inequalities in treatment in these settings.^14^ Data from refugee camps also show high numbers of mental, neurological and substance use problems among refugees living there.^15^ A meta-analysis conducted by Steel *et al* identified exposure to torture and other traumatic events, such as displacement, insecurity and living in refugee camps, as being associated with high rates of PTSD.^7^ With regard to non-quota refugees, European studies have found that the asylum process, particularly long asylum processes, plays a key role as a risk factor for poor mental health among non-quota refugees.^16 17^ Living in institutional accommodation has been found to be associated with poor mental health outcomes among refugees and this association has been attributed to the reduced social and economic prosperity in these settings.^6^ Living in institutional accommodation could both apply to quota refugees in refugee camps and non-quota refugees, who would be moved into accommodation during the asylum process in the country where asylum is sought.

To understand how premigration and migration factors affect refugee mental health we aimed to determine whether risk for NAPD and PTSD differed between quota and non-quota refugees. We hypothesised that the risk of NAPD and PTSD would be different for quota and non-quota refugees, but that region of origin also had an impact of this risk for both groups.^18 19^

## Methods

### Context of quota and non-quota refugees in Sweden

**Figure 1:**
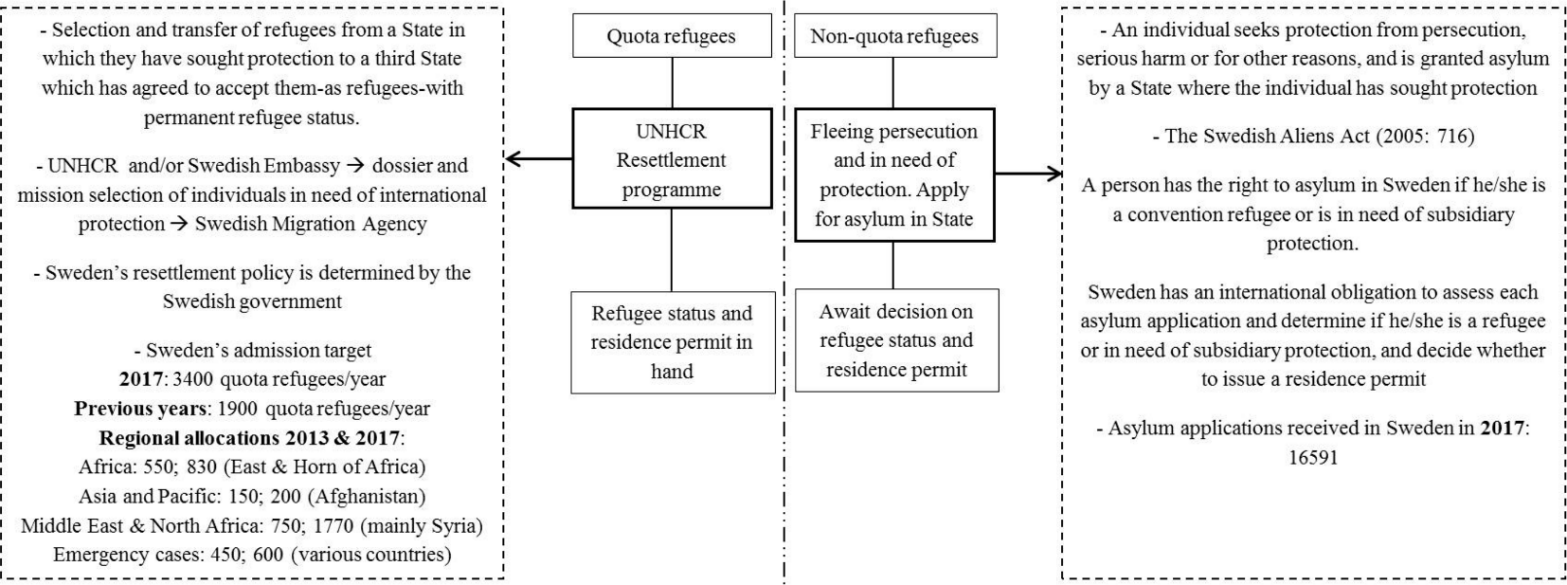
Principal differences involved in the attainment of a refugee status/residence permit among quota and non-quota refugees in Sweden. UNHCR, United Nations High Commissioner for Refugees.

From the 1951 United Nations Refugee Convention, a refugee is documented as someone who ‘owing to well-founded fear of being persecuted for reasons of race, religion, nationality, membership of a particular social group or political opinion, is outside the country of his nationality and is unable or, owing to such fear, is unwilling to avail himself of the protection of that country’.^20^ Sweden’s resettlement programme focuses on individuals in need of international protection^16^ and targets host countries affected by substantial refugee immigration from nearby conflicts, by accepting a specific *quota* of refugees in an attempt to prevent refugee migration from escalating and becoming a permanent issue.^12^ *Non-quota* refugees include refugees who have been seeking asylum and whom have been granted a refugee/subsidiary protection status and residence permits in Sweden.^21^ Figure 1 highlights the process by which both groups of refugees are granted asylum in Sweden.^21^

### Data source

Data for this study were collected from a large, longitudinal database of linked Swedish national registers, known as Psychiatry Sweden (https://ki.se/en/phs/psychiatry-sweden-the-register-linkage-epicss-group), linked via unique personal identification numbers and anonymised by Statistics Sweden.^22^ The Register of Total Population in Sweden (RTB, by Swedish accronym) provided demographic data such as date of birth and sex.^22^ The Immigration and Emigration database (STATIV, by Swedish accronym) provided relevant refugee data such as migrant and refugee subgroup status.^5 23^ Civil status, education in 2010 and disposable income were taken from the Longitudinal Integration Database for Health Insurance and Labour Market Studies (LISA).^24^ For the clinical diagnoses, the national patient registry was used; mortality data were obtained from the Causes of Death register. Swedish registry data are of good quality, the diagnoses have a high validity.^25^

### Study design and population

**Figure 2:**
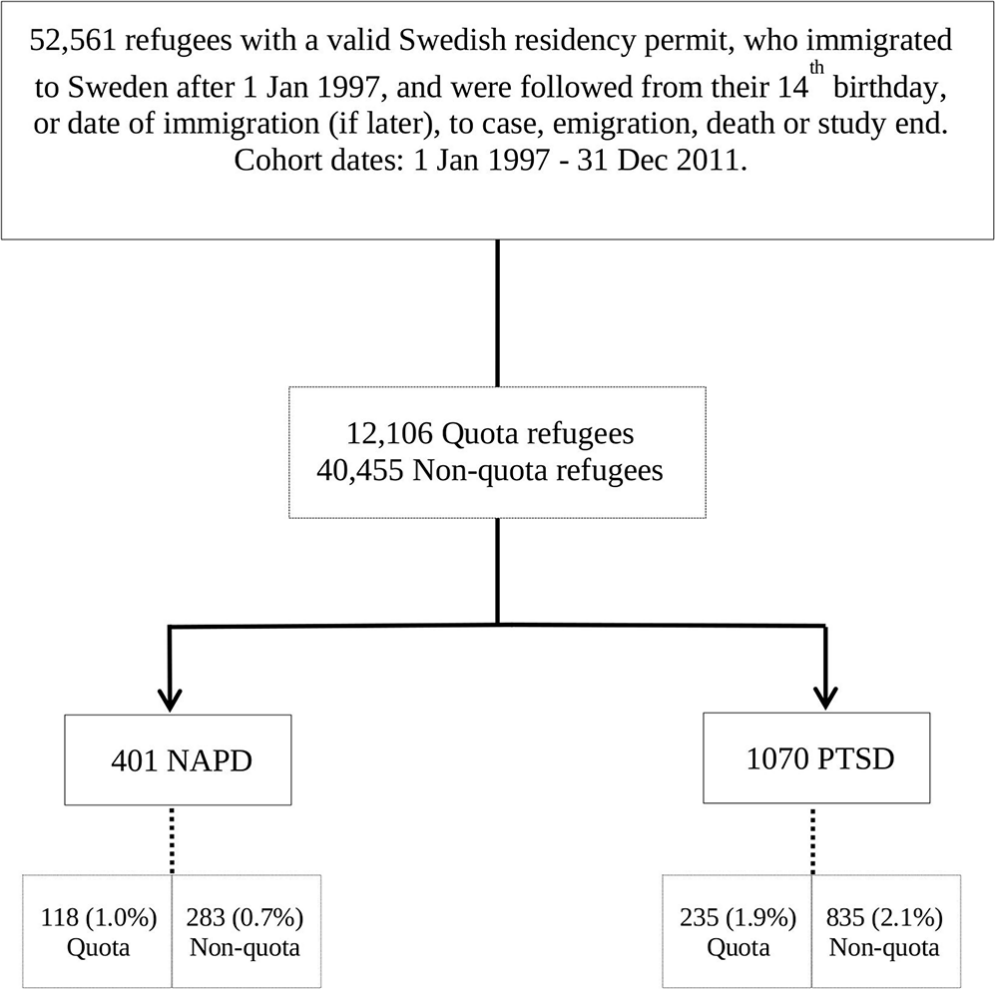
Cohort design. NAPD, non-affective psychotic disorder; PTSD, post-traumatic stress disorder.

A retrospective cohort was established from 1 January 1997 to 31 December 2011. Registered refugees who immigrated to Sweden after 1 January 1997 were followed from their 14th birthday, or date of immigration (if later), up until a diagnosis of NAPD or PTSD, emigration, death or 31 December 2011 (figure 2). The sample size of the cohort was 52 561 refugees who were granted residency in Sweden, in accordance with the Swedish Migration Agency. Individuals without an official residence permit in Sweden, that is, undocumented migrants and persons awaiting an official asylum decision, were omitted from this study.

### Exposures

The primary exposure in this study was *refugee subgroup* status, that is, quota versus non-quota refugee. The reference group throughout the analysis was non-quota refugees.

*Region of origin* was used as a secondary exposure and was defined by country of birth and organised into four principal regions: Asia, Eastern Europe and Russia, Middle East and North Africa, and sub-Saharan Africa.

### Outcomes

The main outcomes were all registered diagnoses from inpatient and outpatient care according to the International Classification of Diseases, Tenth Revision, of NAPD (F20–29) with a high validity^26^ and PTSD (F43.1) also with a high validity.^27^ We defined cases as cohort participants with a first recorded diagnosis between 1 January 1997 and 31 December 2011 in the national patient register, which records diagnoses following inpatient and outpatient admissions in Sweden (including privately run public healthcare settings). Inpatient records are complete since 1987, and complete recording from outpatient settings began in 2001.

### Possible confounders

Possible confounders tested were: birth cohort, sex, region of origin, unaccompanied minors, civil status, education in 2010 and disposable income. These are described in detail as follows:

*Birth cohort* was coded according to birth year into the following categories: 1968–1972, 1973–1977, 1978–1982, 1983–1987, 1988–1992 and 1993–1997.

*Civil status* in 2010 was coded as ‘couple’ for those who were married and ‘single’ to present those who were not married (but could be cohabiting with a partner).

*Education* level was only available for the year 2010 and this variable was coded as follows: ≥9 years (equating to up to 9 years of education), 10–12 years and 12+ years, these categories correspond to the Swedish school system. Statistics Sweden converted the amount of education completed outside Sweden into equivalent levels of schooling in Sweden.

Both civil status and education level had some missing data, and the results presented consider these missing data.

*Age at immigration* was computed from dates of birth and immigration, and was transformed to a nominal variable, with the following categories: 0–4, 5–9, 10–14, 15–19, 20–24, 25–29, 30–34, 35–39 and 40–44. This time did not include waiting time for a residence permit for the non-quota refugee group which is usually between 1 and 24 months.

*Income* in 2010 included individual disposable family income in Sweden, derived from the LISA database. Disposable income, estimated by Statistics Sweden, was defined as annual disposable income based on total family income from all registered sources, including wages, welfare benefits, other social subsidies and pensions. Individual disposable family income is derived by weighting total family income according to household size and composition, with younger children given lower weights than older household members. We defined income as the individual disposable family income transformed into a higher and a lower part with the median separating the two groups based on the total population in 2010.

### Statistical analysis

Descriptive statistics were attained for the cohort and Pearson’s χ^2^ test was used for between-group comparisons for nominal data. Mann-Whitney tests were used to analyse continuous data that were not normally distributed, that is, time to diagnosis and age at diagnosis.

A Cox regression model was fitted to estimate HRs and 95% CIs to investigate the effect of refugee status on risk of developing NAPD and PTSD, after adjusting for birth cohort. A likelihood ratio test was used to test for statistical interaction between refugee subgroup status and sex. If an interaction was found we stratified for sex, else we adjusted for sex. To test for the impact, if any, of the variable’s civil status, education in 2010, and disposable income, Cox regression models including these variables as potential confounders were computed and the HRs were compared with crude HRs.

To determine whether there was a difference in risk of NAPD/PTSD between refugee subgroups according to region of origin, a separate Cox regression model was fitted to control for region of origin, after adjusting for birth cohort. As above a likelihood ratio test was used to test for statistical interaction between refugee subgroup status and sex.

To study the impact, if any, of the potential confounders: civil status, education, age at immigration and income, we included these variables as potential confounders in the Cox regression models. The models including the potential confounders were then compared with the crude HRs. If the potential confounders altered the estimates, they were considered confounders and included in the final model.

Stratification by region of origin was used to examine the effect of this variable as a secondary exposure. The diagnoses NAPD and PTSD were tested in separate models hence some participants could have both outcomes, but not in the same Cox regression model.

Refugees with missing data for civil status and education in 2010 were excluded (n=7299 and n=6733, respectively) and sensitivity analyses were repeated on models excluding the missing data on these variables.

Data processing and statistical analyses were performed using IBM SPSS Statistics for Windows (V.23, IBM).

## Results

Of the 52 561 refugees, quota refugees contributed to 23% of the cohort, while non-quota refugees made up the remaining 77% (see table 1). Non-quota refugees had a significantly higher percentage of male refugees in their cohort compared with quota refugees who displayed a more even distribution of male to female refugees (p<0.001). For both quota and non-quota refugees, the Middle East and North Africa were represented as the most common region of origin for refugees in this cohort (48.6% and 45.1%, respectively). The top source countries or cluster of countries included in each region of origin were the same among both quota and non-quota refugees (see online supplementary table 1). Quota refugees had a higher percentage of refugees aged 15–19 at time of immigration (19.1%) compared with any other age group, whereas age of immigration for non-quota refugees was highest in the 25–29 age group (23.3%). Unaccompanied minors made up nearly a tenth of the non-quota refugee population. A significantly higher percentage of non-quota refugees were shown to have a ‘single’ civil status (60.8%) compared with a ‘couple’ civil status, whereas quota refugees displayed a higher proportion with a ‘couple’ civil status (55.4%) (p<0.001). In total, 118 (1.0%) quota refugees and 283 (0.7%) non-quota refugees were diagnosed with NAPD (p=0.002). However, this significant difference in cumulative risk was not seen between the refugee subgroups for PTSD, for which quota (n=235, 1.9%) and non-quota refugees (n=835, 2.1%) had a similar percentage of diagnoses (p=0.401). For more details about the population, see table 1.

10.1136/jech-2019-212798.supp1 Supplementary data

**Table 1:** Population characteristics by refugee status: quota refugees (quota) and non-quota refugees (non-quota), n and (%), p value for χ^2^ test of differences of proportions between the groups

		Quota n (%)	Non-quota n (%)
	Total	12 106	40 455
NAPD	Yes	118 (1.0)	283 (0.7)
	No	11 988 (99.0)	40 172 (99.3)
PTSD	Yes	235 (1.9)	835 (2.1)
	No	11 871 (98.1)	39 620 (97.9)
Sex	Male	6351 (52.5)	24 798 (61.3)
	Female	5755 (47.5)	15 657 (38.7)
Birth cohort	1968–1972	1720 (14.2)	6134 (15.2)
	1973–1977	1534 (12.7)	7086 (17.5)
	1978–1982	1656 (13.7)	7852 (19.4)
	1983–1987	2150 (17.8)	7703 (19.0)
	1988–1992	2645 (21.8)	6037 (14.9)
	1993–1997	2401 (19.8)	5643 (13.9)
Region of origin	Asia	3025 (25.0)	4529 (11.2)
	Eastern Europe and Russia	536 (4.4)	2965 (6.7)
	Middle East and North Africa	5879 (48.6)	18 248 (45.1)
	Sub-Saharan Africa	2666 (22.0)	14 983 (37.0)
Age at immigration	0–4	494 (4.1)	287 (0.7)
	5–9	1240 (10.2)	1125 (2.8)
	10–14	2080 (17.2)	2753 (6.8)
	15–19	2314 (19.1)	6901 (17.1)
	20–24	1863 (15.4)	7986 (19.7)
	25–29	1887 (15.6)	9426 (23.3)
	30–34	1251 (10.3)	6736 (16.7)
	35–39	809 (6.7)	4449 (11.0)
	40–44	168 (1.4)	792 (2.0)
Unaccompanied minor	Yes	0 (0.0)	3794 (9.4)
	No	12 106 (100.0)	36 661 (90.6)
Disposable income	Lower income	6176 (51.0)	19 546 (48.3)
	Higher income	5930 (49.0)	20 909 (51.7)
Civil status*	Single	4513 (44.6)	20 161 (60.8)
	Couple	5604 (55.4)	12 995 (39.2)
	Total	10 117	33 156
Education in 2010*	≥9 years	3995 (38.4)	12 091 (35.9)
	10–12 years	2787 (26.8)	6594 (19.6)
	12+ years	3617 (34.8)	15 037 (44.6)
	Total	10 399	33 722

*Missing data exist for these variables.

NAPD, non-affective psychotic disorder; PTSD, post-traumatic stress disorder.

Median time to diagnosis for NAPD was documented to be earlier in non-quota refugees (2.0 years, IQR=0.5–4.0) than quota refugees (3.3 years, IQR=1.3–5.8; Mann-Whitney p=0.001) (the median time to diagnoses and median age at first diagnoses are not found in the tables). Median age at first diagnosis for NAPD in quota and non-quota refugees was similar, 28 years (IQR=22–33) and 29 years (IQR=23–35; Mann-Whitney p=0.228), respectively. Median time to diagnosis for PTSD for quota refugees and non-quota refugees was found to be significantly different, with quota refugees experiencing a later diagnosis (2.7 years, IQR=1.1–6.4) compared with non-quota refugees (1.3 years, IQR=0.3–3.5; Mann-Whitney p<0.001). Median age of diagnosis for PTSD for quota refugees was 31 (IQR=24–35), while for non-quota refugees diagnosis age was 30 (IQR=21–35; Mann-Whitney p=0.110) (results not shown in the table).

After stratification by sex, due to identifying statistical interaction between refugee subgroup status and sex (likelihood ratio test; χ^2^=10.4, df=(1), p=0.001), male quota refugees had a significantly higher risk of being diagnosed with NAPD compared with male non-quota refugees (HR=1.41, 95% CI 1.09 to 1.82) while female quota refugees did not display this increased risk (HR=0.65, 95% CI 0.42 to 1.00) (see table 2). All quota refugees (likelihood ratio test; χ^2^=1.7, df=(1), p=0.20) were at a significantly lower risk of developing PTSD compared with non-quota refugees (HR=0.74, 95% CI 0.64 to 0.87). Analyses showed that inclusion of civil status in 2010, education in 2010 and disposable income did not affect the HRs for NAPD or PTSD, therefore they were not included in the final analyses (tables not shown).

**Table 2:** HR and 95% CI for NAPD and PTSD for quota and non-quota refugees

	NAPD	NAPD*		PTSD	PTSD†
		Male HR (95% CI)	Female HR (95% CI)		HR (95% CI)
Non-quota	1	1	1	1	1
Quota	0.99 (0.79 to 1.23)	1.41 (1.09 to 1.82)	0.65 (0.42 to 1.00)	0.69 (0.59 to 0.80)	0.74 (0.64 to 0.86)

*Adjusted for birth cohort.

†Adjusted for birth cohort and sex.

NAPD, non-affective psychotic disorder; PTSD, post-traumatic stress disorder.

After stratification by region of origin, only quota refugees from Asia were at significantly reduced risk of PTSD compared with non-quota refugees from the same region (HR 0.29, 95% CI 0.20 to 0.42). No further difference in risk was evident in any of the other regions (see table 3).

**Table 3:** HR and 95% CI for NAPD and PTSD for quota and non-quota refugees

	n	NAPD	NAPD* HR (95% CI)	n	PTSD	PTSD* HR (95% CI)
Asia						
Non-quota	32	1	1	141	1	1
Quota	17	0.55 (0.30 to 1.00)	0.56 (0.30 to 1.03)	38	**0.27 (0.19 to** **0.39** **)**	**0.29 (0.20 to** **0.42** **)**
Eastern Europe and Russia						
Non-quota	30	1	1	122	1	1
Quota	9	1.46 (0.69 to 3.08)	1.56 (0.74 to 3.31)	15	0.60 (0.35 to1.02)	0.63 (0.37 to 1.09)
Middle East and North Africa						
Non-quota	150	1	1	507	1	1
Quota	68	1.00 (0.74 to 1.33)	1.22 (0.90 to 1.66)	160	**0.72 (0.60 to** **0.86** **)**	0.86 (0.71 to 1.03)
Sub-Saharan Africa						
Non-quota	71	1	1	65	1	1
Quota	24	1.41 (0.87 to 2.27)	1.41 (0.87 to 2.27)	22	1.62 (0.99 to 2.67)	1.44 (0.88 to 1.67)

After stratification for region of origin only quota refugees from Asia had a reduced risk of PTSD compared with non-quota refugees from the same region.

*Adjusted for birth cohort and sex.

NAPD, non-affective psychotic disorder; PTSD, post-traumatic stress disorder.

Sensitivity analyses revealed that exclusion of individuals with missing data for civil status and education in 2010 did not influence the HRs for NAPD or PTSD (tables not shown).

## Discussion

This was the first study to compare the risk of NAPD and PTSD among quota and non-quota refugees. Our findings highlight that male quota refugees had a 41% higher risk of NAPD compared with their non-quota refugee counterparts, while quota refugees, in general, were at 26% lower risk of PTSD compared with non-quota refugees. These results depict a nuanced variance in risk of psychiatric disorders among refugees than previously demonstrated, warranting further research to explain the possible causes behind these effects.

For refugees a multitude of factors linked to the phases of the migration process interplay and this has a cumulative negative effect on their mental health.^28^ Some of these factors include premigration and postmigration stressors such as social and economic adversity, acculturation, isolation, loss of social support and networks.^28^ The experiences of quota and non-quota refugees before and after migration may differ between the two groups.

The migration phase for quota and non-quota refugees is distinctly different. Quota refugees are chosen due to their specific vulnerability. There might be an (ill)health selection among quota refugees as the process of selecting of quota refugees is based on urgency of needs, that is, legal/physical protection, survivors of torture/violence, medical needs, women and girls at risk, family reunification, children/adolescents at risk and when there is no foreseeable alternative.^12^ Non-quota refugees may be at a lower risk for severe psychiatric disorders such as NAPD because only those who are healthy enough are able to make the journey to Sweden. With regard to economic adversity in Sweden, the differences in risk of NAPD and PTSD among refugee groups were not influenced by disposable income, suggesting the two groups are comparable with regard to socioeconomic status. Additionally, inclusion of the postmigration variables education, civil status and year at immigration in the Cox regression model did not alter the estimates either which suggests that these postmigration factors would not explain the results. Time to diagnosis for both NAPD and PTSD was shown to be earlier in non-quota refugees. This was unexpected due to the above described vulnerability among quota refugees and needs further investigation.

Contextual differences in relation to region of origin have not shown to be significant except for the Asia region whereby quota refugees are at a significantly reduced risk of diagnosed PTSD compared with non-quota refugees. One reason for this could be underutilisation of psychiatric care among the Asian refugees as this is seen in Asian migrants in other countries.^24 29 30^

Male quota refugees were at increased risk of NAPD, however this was not seen for female quota refugees which is in line with previous studies.^5^ Non-quota refugees had the highest risk of PTSD with no differences between men and women. This absence of difference between men and women is different from previous findings which show women to have a higher risk of PTSD.^10 11^

### Strengths and limitations

This study has methodological strengths in that it was based on registry data that allowed the retrospective cohort design to be established as well as allowing a Cox regression analysis to be possible. Sweden is a highly suitable setting to conduct this research due to its high-standard official population registers adapted for psychiatric research^25^ and its resettlement policy, established in 1950.^21^

A key limitation of this study is the lack of data on premigration and postmigration experiences and emphasises the need for qualitative studies within this field to have a better understanding of mental illness in refugees and how best to target interventions. Previous qualitative studies with refugees have identified protective factors such as coping mechanisms and resilience.^31^ This is important to consider with regard to quota and non-quota refugees, as qualitative studies may help elucidate their experiences which are not so easy to distinguish from the present study, and further allow a basis to understand why there are differing risks for NAPD and PTSD in these groups. The period covered in this study encompassed the years 1997–2011 as we could only access data until 2011. Since 2011, the refugee humanitarian crisis has escalated, and the results presented are therefore most likely an under-representation of the present-day situation. In addition, the results reflect refugees who sought care and do not consider refugees who did not seek care, that is, incidence versus treated incidence. In addition, the registers are highly complete, recording all psychiatric contacts from inpatient settings from 1987 onwards and from outpatient settings since 2001. Although this may have led to underascertainment from outpatient settings between 1998 and 2000, we have no reason to believe that this would have introduced differential bias by refugee status or region of origin. This is the first study to compare refugee groups by status, without replication and development of the study, there are limited conclusions that can be drawn. Still this paper has practical relevance at two levels. First, as the European Union considers a resettlement programme similar to the quota system^32^ further knowledge of quota refugees is needed. Second, knowledge of the increased risk of mental illness among refugees can help prevention and early interventions, as well as increase knowledge of the aetiology of PTSD and psychosis in the general population.

Future research should focus on epidemiological studies which go beyond descriptive analysis, and instead use analytical and experimental approaches to investigate modifiable risk factors such as premigratory factors (eg, health system in country of origin). Through a better understanding of refugee mental health, there is scope to maximise the impact of interventions which are tailored to refugees.

In conclusion, this paper has shed light on the heterogeneity that exists in refugees in relation to acquiring a refugee status and its complexity surrounding refugee mental health, both of which call for further research to expand our understanding of the mechanisms behind the main findings of this study.

**What is already known on this subject**

- Growing research has revealed that refugees are an ultraexposed subgroup among migrants, more vulnerable to mental illnesses such as post-traumatic stress disorder (PTSD) and non-affective psychotic disorder (NAPD). Quota and non-quota refugees are likely to have experienced different obstacles and stressors along their struggle to get to their refugee status.

**What this study adds**

- Quota and non-quota refugees are at different risk of NAPD and PTSD. Male quota refugees were at an increased risk for NAPD and all quota refugees were at a reduced risk of PTSD compared with non-quota refugees. This research highlights the importance of considering the migration trajectory when trying to understand risk factors for psychiatric disorders among refugees.
