# Supplementary material for: Risk of non-affective psychotic disorder and post-traumatic stress disorder by refugee status in Sweden
Source: J Epidemiol Community Health. 2019 Nov 25;74(3):276–82. doi: 10.1136/jech-2019-212798 (PMC7035697; doi:10.1136/jech-2019-212798)
Supplement: Supplementary data [file jech-2019-212798supp001.pdf]

Supplementary table 1: Countries or cluster of countries included in the regions of origin by refugee status in percent (%).

**Region of origin**		Non-quota %	Quota %
**Asia**	Central Asia	59.3	26.2
	South East Asia	0.6	13.9
**Eastern Europe & Russia**	Russia and the Baltic states	17.6	5.8
	Eastern Europe	5.2	0.8
	Former Yugoslavia	60.7	9.9
**Middle East & North Africa**	Iraq	59.0	15.3
	Iran	6.1	7.4
	Middle East Other	9.2	1.5
	North Africa	1.3	0.2
**Sub-Saharan Africa**	Africa Other	4.5	6.1
	West Africa	0.9	2.0
	Somalia, Eritrea, Ethiopia, Djibouti	79.5	7.0
